# Supplementary material for: Epigenome-Wide Association Analysis of Differentially Methylated Signals in Blood Samples of Patients with Non-Small-Cell Lung Cancer
Source: J Clin Med. 2019 Aug 25;8(9):1307. doi: 10.3390/jcm8091307 (PMC6780065; doi:10.3390/jcm8091307)

**Supplement Figure S1. Epigenome-wide association plot associated with non small cell lung cancer**

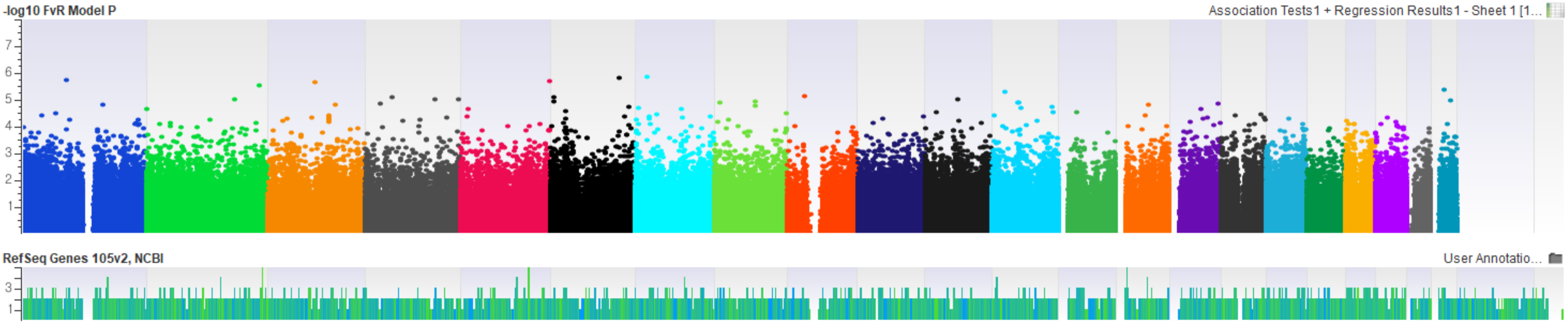


Non small cell lung cancer vs Control

Adjusted for age, sex, smoke (pack years), blood cell type, PCA

**Supplement Figure S2. Epigenome-wide association plot associated with non small cell lung cancer in group of non-smokers**

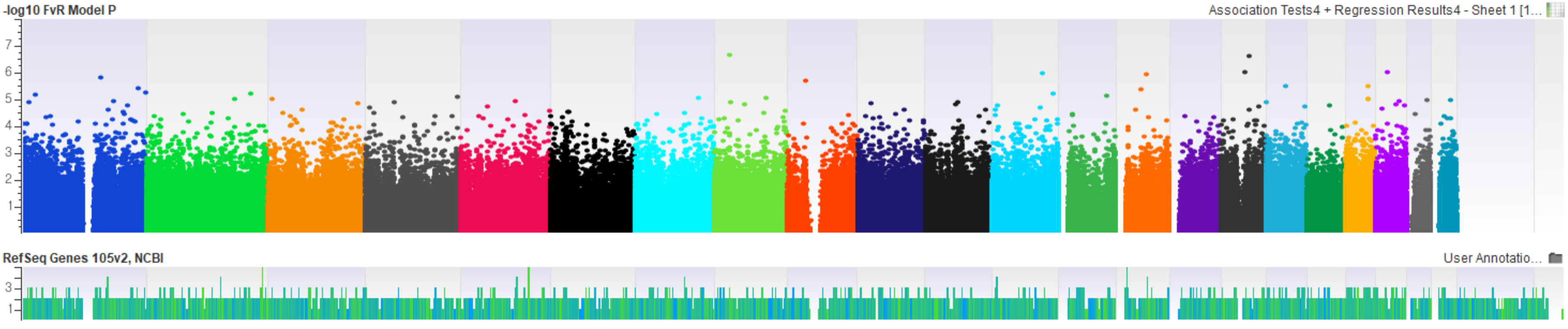


Non small cell lung cancer vs Control in non-smokers

Adjusted for age, sex, smoke (pack years), blood cell type, PCA

**Supplement Figure S3. Epigenome-wide association plot associated with smoking in groups of non small cell lung cancer**

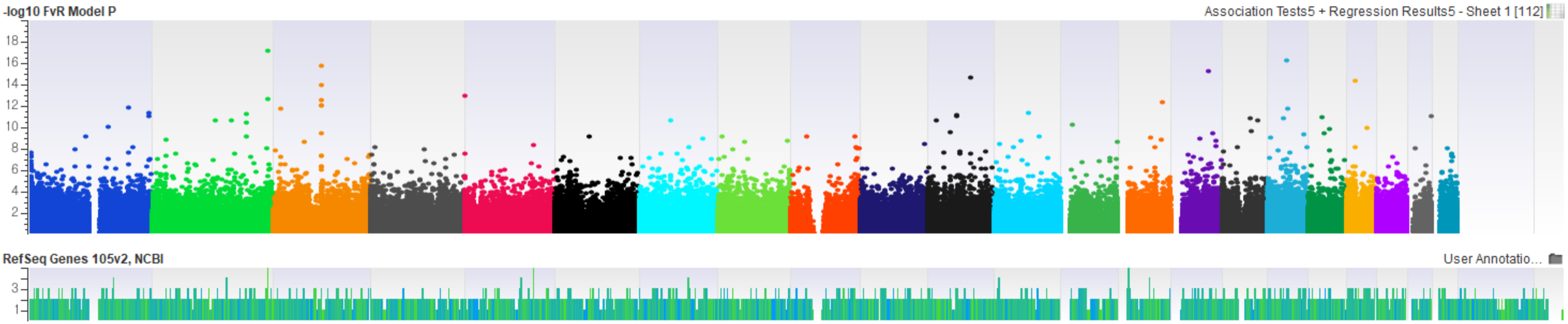


Current smokers vs non-smokers in patients with non small cell lung cancer

Adjusted for age, sex, smoke (pack years), blood cell type, PCA

**Supplement Figure S4. Epigenome-wide association plot associated with smoking in control subjects.**

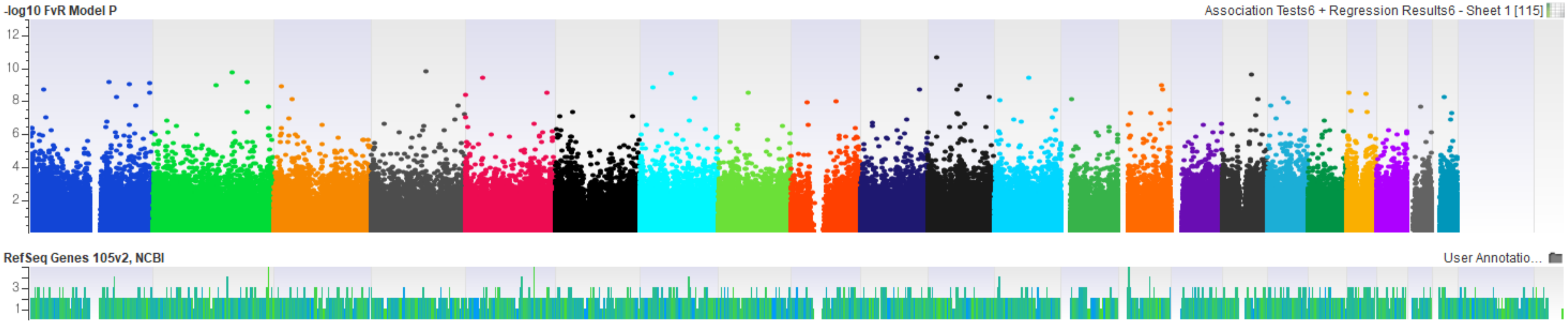


Current smokers vs non-smokers in control

Adjusted for age, sex, smoke (pack years), blood cell type, PCA

**Supplement Figure S5. Correlation of IMP3 methylation with expression in The Cancer Genome Atlas (TCGA) data.**

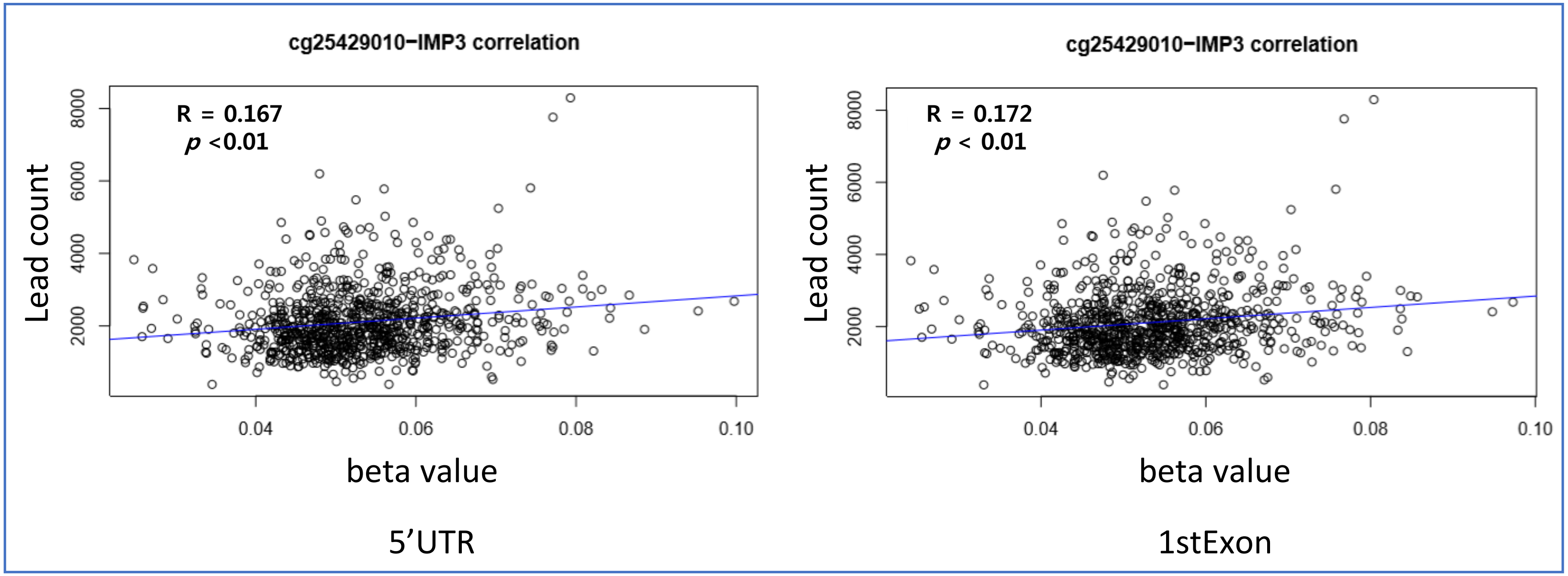

**Supplement Figure S6. Correlation of FAM113B methylation with expression in The Cancer Genome Atlas (TCGA) data.**

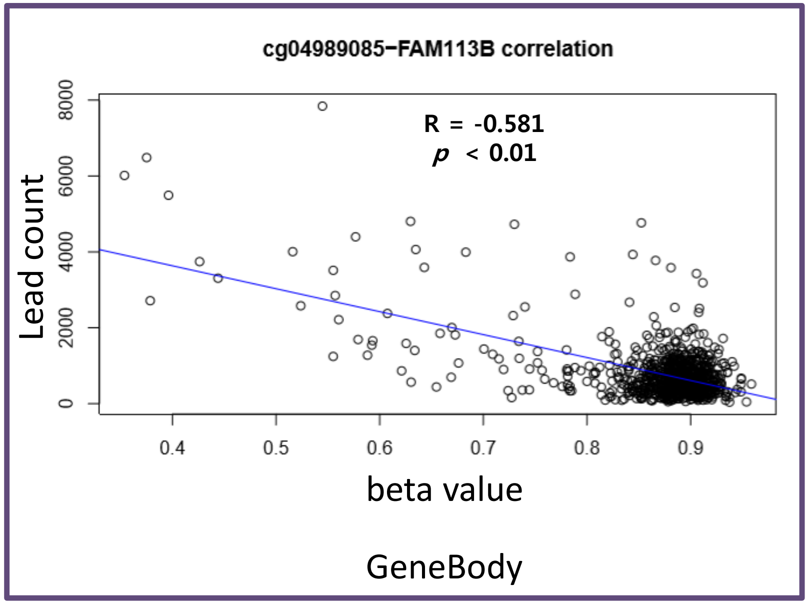

Supplement: Supplementary file 1 [file jcm-08-01307-s001.zip › jcm-572108-supplementary figures.pdf]
