# Supplementary material for: Epigenome-Wide Association Analysis of Differentially Methylated Signals in Blood Samples of Patients with Non-Small-Cell Lung Cancer
Source: J Clin Med. 2019 Aug 25;8(9):1307. doi: 10.3390/jcm8091307 (PMC6780065; doi:10.3390/jcm8091307)
Supplement: Supplementary file 1 [file jcm-08-01307-s001.zip › Supplementary Tables_JCM.pdf]

Table S1. Probe-filtering criteria and summary of the 860k array.
Of the 866,277 CpGs, 695,162 were used for the epigenome-wide association analysis of non-small-cell lung cancer (NSCLC) in our study.

Filtering criteria	CpGs in each criterion	CpGs left in each step
Total CpGs in the 850k array	—	866,277
Probes with a detection p-value of >0.01 in one or more samples	26,884	839,393
Probes with a bead count of <3 in ≥1% of the samples	14,454	824,939
Probes on the X or Y chromosome	17,717	807,222
Probes with SNPs	7,562	799,660
Probes that align to multiple locations	40,832	758,828
Probes overlapping genetic variants at single-base extension sites for Infinium Type I probes	3	758,825
Probes with genetic variants overlapping the body of the probe: 48 base pairs for Infinium Type I probes and 49 base pairs for Infinium Type II probes	63,663	695,162

CpG: cytosine-phosphate-guanine; SNP: single-nucleotide polymorphism.

Table S2. Differentially methylated probes (DMPs), suggestive of an association with NSCLC. (Listed here are 11 CpGs with unadjusted $p < 1.0E-5$.)

Target ID	Chr[a]	Position	Gene ID	Location	Beta[b]	Beta (SE)	*p*-value[c] (adjusted)
cg14988239	7	23349179	C7orf30	3′UTR	−125.782	27.898	1.5E-06
cg14887853	6	139794538	LOC645434	Body	11.052	2.486	1.7E-06
cg02357336	1	87617142	LOC339524	Body	26.903	5.982	1.9E-06
cg05547853	5	180236251	MGAT1	5′UTR	15.296	3.414	2.1E-06
cg19139343	3	97466366	EPHA6	Body	16.222	3.641	2.4E-06
cg18339470	2	228211432	MFF	Body	29.836	6.732	3.1E-06
cg03220797	22	25506763	KIAA1671	Body	−157.885	36.268	4.5E-06
cg06141624	12	24716590	SOX5	TSS1500	71.804	16.779	5.4E-06
cg24468682	9	35690928	TPM2	TSS1500	−571.872	134.670	7.9E-06
cg04497719	6	7961211	BLOC1S5-TXNDC5	Body	−11.021	2.611	8.4E-06
cg02590940	4	52709001	DCUN1D4	TSS1500	147.790	34.829	8.4E-06

[a]Chromosome. [b]Regression coefficient from the statistical models. The covariates age, sex, principal component analysis (PCA), smoking (pack-years), and estimated cell-type proportions were included in the models. Methylation (beta) ranged between 0 and 1 in the analyses. [c]Statistical significance from the statistical model.

Table S3. DMPs, suggestive of an association with NSCLC in current smokers.
(Listed here are 58 CpGs with unadjusted $p < 1.0E-5$.)

Target ID	Chr[a]	Position	Gene ID	Location	Beta[b]	Beta (SE)	*p*-value[c] (adjusted)
cg17970341	11	68139725	LRP5	Body	−57.168	13.400	1.6E-07
cg07900121	6	12539180	—	—	−63.796	15.465	2.8E-07
cg18437361	16	20778121	ACSM3	5′UTR	−559.210	140.060	3.0E-07
cg15571933	22	19186646	CLTCL1	Body	60.071	14.396	3.4E-07
cg12662072	5	63986793	FAM159B	Body	138.297	34.848	3.4E-07
cg19821713	15	96909816	—	—	79.180	19.900	5.7E-07
cg09186006	10	91295299	SLC16A12	1stExon	115.235	29.579	8.7E-07
cg22911013	3	119832292	—	—	−160.392	39.475	8.7E-07
cg03624135	20	9486968	—	—	78.260	19.928	9.2E-07
cg26203839	8	142174631	DENND3	Body	−51.486	12.471	1.1E-06
cg23526861	3	46924956	PTH1R	5′UTR	416.820	106.794	1.1E-06
cg09068724	18	18642613	ROCK1	Body	154.719	38.925	1.2E-06
cg12725760	1	192873912	—	—	22.870	5.761	1.4E-06
cg14453327	7	104845051	SRPK2	Body	−365.463	92.396	1.6E-06
cg25947890	9	139403372	NOTCH1	Body	−61.560	15.947	1.7E-06
cg00353531	9	93805672	—	—	57.619	14.576	1.8E-06
cg16032065	9	120993167	—	—	−26.422	6.626	1.8E-06
cg15512166	5	179544005	RASGEF1C	Body	53.703	13.898	2.1E-06
cg05366909	11	125333752	FEZ1	Body	−56.155	14.523	2.1E-06
cg27176357	19	57702500	ZNF264	TSS1500	23.183	5.996	2.4E-06
cg17186373	8	74884456	TCEB1	TSS200	−694.001	172.515	2.6E-06
cg01881064	1	54396087	HSPB11	Body	−403.460	101.755	2.7E-06
cg14430698	2	131482665	—	—	−51.301	12.679	2.7E-06
cg11074192	13	78494442	EDNRB	TSS1500	317.094	83.841	2.8E-06
cg05478330	15	78189752	—	—	−105.414	27.167	2.9E-06
cg07538771	5	77458683	AP3B1	Body	−154.489	39.730	3.0E-06
cg00521863	2	171783942	—	—	186.292	48.311	3.0E-06
cg02642363	22	37422281	MPST	Body	−225.764	56.457	3.1E-06
cg22187630	19	13616871	CACNA1A	1stExon	63.472	16.325	3.1E-06
cg05425050	11	27494410	LGR4	TSS200	1,009.760	263.918	3.6E-06
cg12080687	2	47063626	LOC100134259	Body	55.540	14.221	3.8E-06
cg20414505	1	6061279	—	—	162.857	43.825	3.8E-06
cg22227726	1	202939745	—	—	−32.005	8.043	3.8E-06
cg07654631	3	128295655	LINC01565	TSS1500	58.898	15.527	4.0E-06

cg24364143	1	33005702	ZBTB8A	5′UTR	497.461	133.996	4.2E-06
cg08217716	10	102589250	PAX2	3′UTR	314.549	79.506	4.6E-06
cg24433265	12	52389247	ACVR1B	3′UTR	−55.661	14.121	4.7E-06
cg11638352	15	85526469	PDE8A	5′UTR	−57.437	14.633	5.2E-06
cg19834134	7	82791675	PCLO	1stExon	117.412	31.853	5.4E-06
cg19668476	4	153506267	—	—	−39.265	10.357	5.6E-06
cg04618230	4	54677002	—	—	48.618	12.592	5.6E-06
cg09146645	6	39186104	KCNK5	Body	−49.755	12.929	5.6E-06
cg01411440	17	7835277	TRAPPC1	5′UTR	−559.302	144.065	6.2E-06
cg07523753	1	2979582	FLJ42875	Body	37.189	10.095	6.4E-06
cg08115833	20	9048763	—	—	83.563	21.924	6.5E-06
cg14998959	10	134564163	INPP5A	Body	−477.661	124.111	7.1E-06
cg09890891	15	96904723	—	—	72.062	18.739	7.4E-06
cg26000663	11	26353723	ANO3	1stExon	50.896	13.807	7.8E-06
cg07740710	16	660965	RAB40C	Body	−81.036	20.848	7.9E-06
cg09855384	17	35684812	ACACA	Body	−205.782	54.722	8.1E-06
cg01999118	3	35465004	—	—	181.117	48.043	8.6E-06
cg13138155	1	92950575	GFI1	5′UTR	−1,268.146	339.832	8.8E-06
cg21741923	2	4274538	—	—	−91.538	25.071	9.1E-06
cg04390191	2	231090628	SP110	TSS200	−51.041	13.702	9.2E-06
cg09090353	11	67194923	RPS6KB2	TSS1500	64.876	17.342	9.5E-06
cg13043696	10	1917044	—	—	−35.978	9.907	9.5E-06
cg22515201	22	38577827	PLA2G6	TSS200	−847.118	220.586	9.7E-06
cg16995791	1	59703067	—	—	26.072	7.025	9.8E-06

[a]Chromosome. [b]Regression coefficient from the statistical models. The covariates age, sex, PCA, smoking (pack-years), and estimated cell-type proportions were included in the models. Methylation (beta) ranged between 0 and 1 in the analyses. [c]Statistical significance from the statistical model.

Table S4. DMPs, suggestive of an association with NSCLC in nonsmokers.
(Listed here are 19 CpGs with unadjusted $p < 1.0E-5$.)

TargetID**Target ID**	**Chr**[a]	**Position**	**Gene ID**	**Location**	**Beta**[b]	**Beta (SE)**	P***p*-value**[c] **(adjusted)**
ch.8.745965F	8	30420648	RBPMS	3′UTR	−−530.022	118.417	2.4E-07
cg01834111	16	57085391	NLRC5	Body	99.248	22.487	2.7E-07
cg15328849	16	49623272	ZNF423	Body	−−39.089	9.049	1.0E-06
cg11793257	20	25218719	.—	.—	43.783	10.341	1.0E-06
cg13015171	12	102515051	PARPBP	5′UTR	34.287	8.198	1.2E-06
cg12717745	14	61852241	PRKCH	Body	−−52.909	12.574	1.2E-06
cg16196595	1	156192737	PMF1	Body	30.176	7.091	1.6E-06
cg02300714	9	37800774	DCAF10	TSS200	−−832.535	202.881	2.1E-06
cg00550617	17	39405817	KRTAP9-9	Body	−−20.114	4.868	3.4E-06
cg05098732	19	46367776	FOXA3	1stExon	55.774	13.776	3.5E-06
cg02414710	1	232650142	SIPA1L2	1stExon	−−153.876	37.527	4.1E-06
cg05453127	14	50439817	.—	.—	49.111	11.865	4.4E-06
cg13910601	1	247711035	GCSAML	TSS1500	52.147	13.505	6.2E-06
cg11926456	2	211432972	CPS1	Body	−−25.281	6.389	6.5E-06
cg23786360	12	123487931	PITPNM2	Body	−−46.662	11.519	6.6E-06
cg13296912	1	24208741	CNR2	5′UTR	−−206.904	51.616	7.2E-06
cg13895393	13	97928122	MBNL2	5′UTR	277.852	69.824	7.8E-06
cg16433265	4	186741815	SORBS2	5′UTR	−−64.968	16.297	8.4E-06
cg23877147	8	103897520	AZIN1-AS1	Body	321.986	81.947	9.5E-06

[a]Chromosome. [b]Regression coefficient from the statistical models. The covariates age, sex, PCA, smoking (pack-years), and estimated cell-type proportions were included in the models. Methylation (beta) ranged between 0 and 1 in the analyses. [c]Statistical significance from the statistical model.

Table S5. DMPs significantly associated with smoking in patients with NSCLC.
(Listed here are the top 30 CpGs with $p < 7.2E\text{-}08$ after Bonferroni's correction).

Target ID	Chr[a]	Position	Gene ID	Location	Beta[b]	Beta (SE)	P*p*-value[c] (adjusted)
cg21566642	2	233284661	.—	.—	−104.674	32.691	9.2E-18
cg17739917	17	38477572	RARA	5′UTR	−109.959	37.629	7.1E-17
cg19859270	3	98251294	GPR15	1stExon	−291.857	103.043	2.1E-16
cg18110140	15	75350380	.—	.—	−105.341	30.213	7.9E-16
cg14391737	11	86513429	PRSS23	5′UTR	−70.984	18.954	2.8E-15
cg03636183	19	17000585	F2RL3	Body	−65.149	16.567	6.0E-15
cg04180924	3	98272064	.—	.—	−333.702	88.374	1.5E-14
cg05934812	5	334322	AHRR	Body	−106.725	27.603	1.4E-13
cg01940273	2	233284934	.—	.—	−76.951	18.926	2.7E-13
cg02978227	3	98292027	.—	.—	−130.243	35.506	3.7E-13
cg02325951	14	89878619	FOXN3	Body	72.205	17.665	5.8E-13
cg08064403	3	98240258	CLDND1	Body	−95.592	24.545	1.1E-12
cg18754985	3	98237750	CLDND1	Body	−156.470	38.314	1.3E-12
cg26919182	1	202522232	PPP1R12B	Body	−13.713	3.375	1.8E-12
cg11643285	3	16411667	RFTN1	Body	−31.382	7.824	2.2E-12
cg12607525	17	42286849	UBTF	Body	63.698	15.611	2.3E-12
cg15228509	1	243074717	.—	.—	−27.236	6.432	5.2E-12
cg09516963	12	68042445	DYRK2	TSS200	−19.032	4.187	6.2E-12
cg06759085	2	191555076	NAB1	3′UTR	90.403	24.341	6.6E-12
cg04858776	11	59318494	.—	.—	−78.538	19.401	9.3E-12
cg17232883	11	59318136	.—	.—	−141.818	34.167	1.0E-11
cg23283320	21	48055893	PRMT2	5′UTR	−359.576	89.187	1.1E-11
cg12691488	1	243053673	.—	.—	37.159	8.505	1.2E-11
cg25304146	18	30092971	WBP11P1	Body	61.927	14.338	1.6E-11
cg06152526	16	57290525	PLLP	3′UTR	70.146	16.955	1.7E-11
cg03218192	17	33914403	AP2B1	1stExon	−73.386	17.419	1.9E-11
cg03226871	2	128128958	.—	.—	44.857	10.064	2.5E-11
cg03626220	16	71052200	HYDIN	Body	46.804	10.836	2.6E-11
cg16021537	2	161349986	RBMS1	1stExon	−111.382	26.939	2.8E-11
cg06513015	7	64459246	ERV3-1	5′UTR	−33.465	7.795	2.8E-11

[a]Chromosome. Regression coefficient from the statistical models. The covariates age, sex, PCA, smoking (pack-years), and estimated cell-type proportions were included in the models. Methylation (beta) ranged between 0 and 1 in the analyses. [c]Statistical significance from the statistical model.

Table S6. DMPs significantly associated with smoking in control subjects.
(Listed here are the top 30 CpGs with $p < 7.2E\text{-}08$ after Bonferroni's correction).

Target ID	Chr[a]	Position	Gene ID	Location	Beta[b]	Beta (SE)	***p*-value**[c] **(adjusted)**
cg04322608	11	17392359	NCR3LG1	Body	65.941	15.732	2.5E-11
cg22266749	4	110223959	COL25A1	TSS200	50.586	12.345	1.6E-10
cg16021537	2	161349986	RBMS1	1stExon	−109.289	28.632	1.9E-10
cg06513015	7	64459246	ERV3-1	5′UTR	−37.561	10.024	2.3E-10
cg04946709	16	59789030	LOC644649	Body	54.463	14.027	2.4E-10
cg09516963	12	68042445	DYRK2	TSS200	−20.358	5.175	3.9E-10
cg10546176	5	34929404	DNAJC21	TSS1500	−200.599	51.693	4.2E-10
cg03618918	1	160865097	—	—	39.608	9.706	7.2E-10
cg20262915	2	191524489	NAB1	Body	53.828	13.307	7.6E-10
cg15228509	1	243074717	—	—	−26.545	6.572	8.5E-10
cg26919182	1	202522232	PPP1R12B	Body	−11.553	2.772	9.4E-10
cg11984238	14	88965446	PTPN21	Body	−108.748	27.704	1.1E-09
cg12052203	11	66115045	B3GNT1	1stExon	29.707	6.875	1.2E-09
cg03226871	2	128128958	—	—	46.551	11.520	1.2E-09
cg11643285	3	16411667	RFTN1	Body	−24.991	5.886	1.3E-09
cg10739556	7	27192056	—	—	−117.474	29.665	1.5E-09
cg17232883	11	59318136	—	—	−122.665	30.678	2.0E-09
cg10292832	1	26798740	HMGN2	TSS200	−126.620	30.577	2.0E-09
cg14030268	10	119135296	PDZD8	TSS1500	−837.031	240.800	2.1E-09
cg02325951	14	89878619	FOXN3	Body	54.972	14.030	2.2E-09
cg12691488	1	243053673	—	—	34.551	8.680	3.1E-09
cg25849844	5	164386021	—	—	206.729	57.117	3.1E-09
cg11050859	8	60031823	TOX	TSS200	−115.158	29.343	3.2E-09
cg19388739	19	3581993	—	—	136.665	36.582	3.3E-09
cg01276128	19	39138334	ACTN4	5′UTR	−365.038	94.212	4.1E-09
cg05575921	5	373378	AHRR	Body	−20.761	4.774	4.6E-09
cg25429619	1	174846521	RABGAP1L	5′UTR	35.506	9.444	5.6E-09
cg14095048	22	25342876	.	.	91.063	22.136	5.7E-09
cg04224661	11	123375288	—	—	69.038	17.376	6.0E-09
cg22108930	7	111800870	DOCK4	Body	−78.791	20.691	6.9E-09

[a]Chromosome. [b]Regression coefficient from the statistical models. The covariates age, sex, PCA, smoking (pack-years), and estimated cell-type proportions were included in the models. Methylation (beta) ranged between 0 and 1 in the analyses. [c]Statistical significance from the statistical model.

Table S7. DMPs, suggestive of an association with stage I NSCLC.
(Listed here are 15 CpGs with unadjusted $p < 1.0E-5$)

Target ID	Chr[a]	Position	Gene	Location	Beta[b]	Beta (SE)	*p*-value[c]
cg08135765	4	6996753	TBC1D14	Body	−225.3	49.5	3.1E-07
cg00389714	12	58731100	—	—	−29.7	7.0	1.3E-06
cg17324327	7	43622228	STK17A	TSS1500	−650.9	155.8	2.2E-06
cg09171428	22	44584443	PARVG	Body	75.4	18.5	2.4E-06
cg21808045	17	75463984	SEPT9	Body	38.5	9.4	2.9E-06
cg05860195	16	89324033	—	—	−38.9	9.7	3.2E-06
cg12561658	12	126579383	LOC101927464	TSS1500	20.9	5.1	3.5E-06
cg06640763	7	149473097	SSPO	TSS200	172.5	41.5	5.1E-06
cg26600802	10	115939115	TDRD1	5′UTR	−35.9	8.5	5.2E-06
cg06015439	12	91669322	—	—	34.8	8.6	6.6E-06
cg05047465	10	27793441	RAB18	Body	440.6	108.2	8.6E-06
cg02238928	20	55837591	BMP7	Body	20.1	4.9	9.7E-06
cg16391151	7	30518197	NOD1	5′UTR	−938.9	234.8	9.8E-06
cg18375700	17	42975879	CCDC103	TSS1500	49.5	12.9	9.9E-06
cg09876271	14	55595895	LGALS3	TSS200	−305.5	75.8	1.0E-05

[a]Chromosome.
[b]Regression coefficient from the statistical models. The covariates age, sex, PCA, smoking (pack-years), and estimated cell-type proportions were included in the models. Methylation (beta) ranged between 0 and 1 in the analyses.
[c]Statistical significance from the statistical model.
